# Physician Attitudes and Perceptions of Complementary and Alternative Medicine (CAM): A Multicentre Italian Study

**DOI:** 10.3389/fonc.2020.00594

**Published:** 2020-04-28

**Authors:** Massimiliano Berretta, Luca Rinaldi, Rosaria Taibi, Paolo Tralongo, Alberto Fulvi, Vincenzo Montesarchio, Giordano Madeddu, Paolo Magistri, Sabrina Bimonte, Marco Trovò, Patrizia Gnagnarella, Arturo Cuomo, Marco Cascella, Arben Lleshi, Guglielmo Nasti, Sergio Facchini, Francesco Fiorica, Raffaele Di Francia, Giuseppe Nunnari, Giovanni Francesco Pellicanò, Aurelio Guglielmino, Marco Danova, Sabrina Rossetti, Alfonso Amore, Anna Crispo, Gaetano Facchini

**Affiliations:** ^1^Department of Medical Oncology, Istituto Nazionale Tumori, IRCCS - CRO, Aviano (PN), Italy; ^2^Department of Advanced Medical and Surgical Sciences, University of Campania “Luigi Vanvitelli”, Naples, Italy; ^3^Division of Medical Oncology, “Umberto I” Hospital, Siracusa, Italy; ^4^Division of Medical Oncology, “Gemelli” Hospital, Roman, Italy; ^5^Division of Medical Oncology, “Monaldi” Hospital, Naples, Italy; ^6^Division of Infectious Diseases, University of Sassari, Sassari, Italy; ^7^Department of Surgery, University of Modena and Reggio Emilia, Modena, Italy; ^8^Department of Anaesthesia and Pain Medicine, Istituto Nazionale Tumori “Fondazione G. Pascale” IRCCS, Naples, Italy; ^9^Division of Radiotherapy, “Santa Maria della Misericordia” Hospital, Udine, Italy; ^10^Division of Epidemiology and Biostatistics IEO European Institute of Oncology IRCCS, Milan, Italy; ^11^Division of Medical Oncology B, Istituto Nazionale Tumori “Fondazione G. Pascale” IRCCS, Naples, Italy; ^12^Department of Urology, University of Naples “Federico II”, Naples, Italy; ^13^Division of Radiotherapy, “Mater Salutis” Hospital, Legnago, Italy; ^14^Gruppo Oncologico Ricercatori Italiani, GORI, Pordenone, Italy; ^15^Division of Infectious Disease, University of Messina, Messina, Italy; ^16^Division of Anaesthesia, Policlinico Universitario, University of Catania, Catania, Italy; ^17^Department of Internal Medicine and Medical Oncology, Vigevano Civic Hospital, ASST of Pavia, Vigevano, Italy; ^18^Medical Oncology, Department of Uro-Gynaecological Oncology 'Istituto Nazionale Tumori' 'Fondazione G. Pascale' IRCCS, Naples, Italy; ^19^Division of Surgery Melanoma and Skin Cancer, 'Istituto Nazionale Tumori' 'Fondazione G. Pascale' IRCCS, Naples, Italy; ^20^Unit of Epidemiology, 'Istituto Nazionale Tumori' 'Fondazione G. Pascale' IRCCS, Naples, Italy

**Keywords:** complementary medicine, alternative medicine, physicians, cancer, treatment, Italian survey, attitudes

## Abstract

**Purpose:** Complementary and Alternative Medicine (CAM) interventions are widely used by patients with chronic disorders, including cancer, and may interact with cancer treatment. Physicians are often unaware of this, probably due to poor patient-physician communication on CAM. The purpose of this study was to evaluate physicians' knowledge, attitudes and practice patterns regarding CAM in a survey conducted in Italy.

**Methods:** A questionnaire was administered to 438 physicians (11 Italian hospitals) who predominantly treat patients with chronic disease, to collect personal and professional data and information on attitudes toward CAM and its possible role in Conventional Medicine (CM).

**Results:** Of the 438 participants, most were specialists in oncology (18%), internal medicine (17%), surgery (15%), and radiotherapy (11%). Most worked at university (44%) or research hospitals (31%). Forty-two percent of participants believed that CAM could have an integrative role within CM. Oncologists were the physicians who were best informed on CAM (58%). Physicians working at research institutes or university hospitals had a greater knowledge of CAM than those employed at general hospitals (*p* < 0.0001), and those who were also involved in research activity had a greater knowledge of CAM than those who were not (*p* < 0.003). Length of work experience was significantly related to CAM knowledge. Moreover, 55% of participants suggest CAM interventions to their patients and 44% discuss CAM with them. The best-known interventions were acupuncture, *Aloe vera* and high-dose vitamin C.

**Conclusion:** CAM use by patients with chronic disease and/or cancer has become a topical issue for the scientific community and for physicians. Knowing the reasons that prompt these patients to use CAM and guiding them in their decisions would improve treatment and outcomes and also benefit healthcare systems. Our findings contribute to a greater understanding of CAM knowledge, attitudes, and practice among Italian physicians. Further research is needed to identify the more effective CAM treatments and to work toward an integrated healthcare model.

## Introduction

According to the U.S. National Center for Complementary and Integrative Health (NCCIH), Complementary and Alternative Medicine (CAM) therapies include a wide spectrum of practices and products, either biological (e.g., herbs or botanicals, vitamins, minerals, probiotics, homeopathic products, and Chinese herbal remedies) or non-biological (e.g., prayer, meditation, music therapy, yoga). These interventions are defined as “alternative” when they are used *instead of* Conventional Medicine (CM) and as “complementary” when they are used *together with* it (1). Their popularity has been increasing, and according to 26 studies conducted all over the world by the 1990s they were used by 7–64% of patients with chronic disorders, including cancer (2–5). In the past decade the interest in CAM has grown further, the main reasons being massive internet marketing, dissatisfaction with CM, and a desire by patients to achieve greater control over medical decisions (2).

CAM has become widespread in most industrialized countries; individuals who have used it at least once account for about 70% of the population in Canada (6), ~50% in Italy, France and Australia (7–9), 40% in the USA (3), 30% in Japan (2), and 31% in Belgium (8).

The diffusion of CAM therapies is relevant to physicians, because several biologically based approaches, such as herbs and supplements, can interfere with CM treatment efficacy, including antiblastic chemotherapy (AC) and target therapy (TT), besides heightening the risk of treatment-related toxicity and other complications. For example, St John's wort, Asian ginseng and green tea have all been found to induce toxicity and to interact with a number of medications, including AC and TT (10–13). A study of adult cancer patients estimated that 28% were at risk of AC-herb interactions; notably, 46% of these patients were treated with curative intent (14). The interactions described between the most common AC and CAM interventions published in the English literature are reported in Table 1 (15–42). To the best of our knowledge, there are no studies in English on interactions between immunotherapy and CAM.

**Table 1:** Most common CAM interventions adopted by cancer patients and possible interactions with AC.

**CAM agents**	**Metabolic pathway**	**Interaction with cancer treatments**	**Adverse events**	**Reference**
Active hexose-correlated compound isolated from shiitake mushrooms	CYP2D6 induction	May reduce the activity of ADM, which is a substrate of this enzyme, and of AIs	Diarrhea and itching	(15)
Ananas Pineapple (bromelain)	CYP2C9 inhibition	Risk of overdosage in patients treated with TXL	Exacerbation of hand and foot syndrome	(16)
β-carotene		Alcohol consumption has an adverse effect on β-carotene activity	The hepatotoxic effects of ethanol may be potentiated by high-dose β-carotene	(17)
B-elemene (terpene from *Rhizoma zedoariae* and mint)		Increased DDP and taxane activity	No adverse events recorded	(18)
Bitter melon (*Momordica charantia*)	P-gp and CYP2C9 inhibition	Increased intracellular concentration VBL and TXL	No adverse events were recorded	(19)
Turmeric (*Curcuma* longa)	Weak CYP1A2, CYP2B6, CYP2C9, and CYP2D6 inhibition	Risk of overdosage in patients treated with bendamustine and inefficacy of prodrugs (CTX, TAM)	Allergic dermatitis and bile duct obstruction	(20)
Cannabinoids	CYP2C9 induction	Risk of overdosage in patients treated with prodrugs (CTX, TAM)	Gastrointestinal complaints^∧^	(21)
Di Bella multitherapy§	GH inhibition, enhances IGF-binding protein-1 secretion	The opioid antagonist properties of somatostatin reduce the analgesic effect of opioids in patients with advanced cancer	Gastrointestinal complaints^∧^, cholelithiasis, and hyperglycaemia	(22)
*Echinacea*	Potent CYP3A4 inhibition	Improved pharmacokinetics of CTX, DAS, TXT, ERL, IMT, SOR (weak) VALK (high), and VP16	Severe thrombocytopaenia in a patient receiving VP16	(23)
Essiac^*^	CYP3A4 inhibition	Risk of overdosage in patients treated with BTZ, DAS, TXT, ERL, IMT, SOR, VALK	Gastrointestinal complaints^∧^	(24)
Folic acid	MTHFR-enhancing activity	Improved activity of antimetabolite drugs (5-Fu)	Concurrent use of folic acid may antagonize the effects of certain anticonvulsants	(25)
Glucans from mushrooms^°^	EGFr and mTOR inhibition	May antagonize TAM in patients with estrogen-positive breast cancer	Immunosuppressive effects	(26)
Green tea	CYP3A4 inhibition	Similar to Essiac	High ALT levels	(27)
*Gingko biloba*	CYP3A4 CYP2C19, P-gp	Similar to Essiac	Nervousness	(28)
Ginseng	CYP3A4 inhibition	Increased risk of IMT hepatotoxicity	High ALT levels	(29)
Glutathione	GSH, GSTP1	Increased AC detoxification	Mucosal hypersecretion	(30)
Grapefruit (including juice)	CYP3A4 inhibition	Not recommended during ADM due to oxidations	Gastrointestinal complaints^∧^	(31)
Liquorice	weak CYP2B6, CYP3A4 inhibition	Similar to Essiac (weak)	Hypertension, retinopathy and nephropathy	(32)
Milk thistle	Weak CYP2C8 and CYP2C9 inhibition	Risk of overdosage in patients taking CTX, TXL	No adverse events recorded	(33)
Oleander	P-gp and mTOR inhibition	May increase the blood levels of substrate drugs such as TKIs.	Gastrointestinal complaints^∧^	(34)
Omega 3	p53	Reduces platin activity	Platin-drug resistance	(35)
Ozone therapy	ND	Not recommended during ADM due to oxidation	ND	(36)
Quercetin	Strong CYP3A4 and CYP2C19 inhibition	Similar to Essiac	High ALT levels	(37)
Resveratrol	CYP3A4, CYP2D6, CYP2C9, inhibition	Protective effects against DDP- and ADM-induced cardiotoxicity, due to upregulation of SIRT1-mediated p53 deacetylation	No adverse events recorded	(38)
Spirulina and blue-green algae	CYP 1A2 and 2E1 inhibitions	Induces accumulation of drugs metabolized by these enzymes, including bendamustine	Increases the risk of their side effects	(39)
St. John's worth (*Hypericum*)	CYP3A4 induction	Improved CTX, DAS, TXT, ERL, IMT, SOR, and VALK pharmacokinetics	Headache, dry mouth, sleepiness, gastrointestinal complaints^∧^	(40)
Vitamin C	ND	May reduce the effectiveness of VCR, ADM, MTX, DDP, BTZ, IMT	Kidney stones	(41)
Zeolite	Protein kinase B inhibition	Enhances the effect of ADM due to its antioxidant properties	Pulmonary fibrosis, leucocytosis	(42)

AC, Antiblastic chemotherapy; ADM, Doxorubicin; ALT, Alanine aminotransferase; BTZ, Bortezomib; CTX, Cyclophosphamide; CYP, Cytochrome P450; DAS, Dasatinib; DDP, Cisplatin; ERL, Erlotinib; 5-FU, Fluorouracil; IA, Aromatase inhibitors; IMT, Imatinib; MTX, Methotrexate; NA, Not available; ND, Not documented; P-gp, P-glycoprotein; SOR, Sorafenib; TAM, Tamoxifen; TXL, Paclitaxel; TXT, Docetaxel; VALK, Vinca alkaloids; VBL, Vinblastine; VCR, Vincristine; VP16, Etoposide.

***Source:**http://reference.medscape.com/drug-interactionchecker and Memorial Sloan Kettering Cancer Center: https://www.mskcc.org/cancer-care/integrative-medicine/herbs/ginseng-asian#references-24*.

* Herbal mixture patented as a cancer therapy by Rene Caisse in 1920 in Canada.

° Grifula frondosa (maitake), Lentinula edodes (shiitake), Ganoderma lucidum (reishi), etc.

§ Somatostatin, Bromocriptine, Fluvoxamine, Melatonin.

∧ *Gastrointestinal complaints: diarrhea, vomiting, and nausea*.

The wide diffusion of CAM and the attendant risk for some patients—especially those receiving active anticancer treatment (ACT)—involve that physicians should inquire about their use by patients and be familiar with the more common CAM therapies.

In a recent multicentre Italian study (7), we found that 49% of cancer patients combined CAM remedies with their ACT and that in 67% of cases the interventions were self-prescribed. Their main sources of information were the internet and the media (48%), whereas only 6% of patients received information on CAM from physicians. Critically, 85% of patients were not aware of the risk of side-effects of CAM remedies and of potential interactions with CM treatments. The latter issue raises disturbing questions and highlights the need for greater patient-physician communication on CAM. Although oncologists generally discuss treatment options with patients (choice of treatment, therapeutic targets, side-effects), they largely ignore CAM (43–45). A study conducted at the University of Texas MD Anderson Cancer Center in Houston has found limited communication and discordant views among physicians with regard to CAM therapies (46). Insufficient patient-oncologist communication on CAM has also been reported (46). Poor communication between healthcare professionals and patients has been described with regard to CAM; for instance, in a previous Italian multicentre survey, Crocetti et al. (47) highlighted a poor attitude of oncologists toward CAM. According to data published by Censis (an Italian socioeconomic research body) on fake news on medications in 2017, 28% of Italians who have a medical problem consult primarily “Dr Google,” likely due to poor or no communication with their physicians (48).

The medical education of Italian physicians is evidence-based. Most have never been taught CAM at any stage of their training, a fact that may be ascribed to lack of significant scientific evidence for its effectiveness. Indeed, the current literature on CAM and cancer is largely based on the patients' standpoint, whereas papers addressing the physicians' point of view are now beginning to be published. Since the attitudes toward CAM of Italian physicians who treat patients with chronic disorders, including cancer, have never been surveyed, we set out to investigate the personal and professional characteristics and CAM attitudes, knowledge, and use in a sample of physicians who predominantly treat this type of patients.

## Materials and Methods

### Participants

A nationwide cross-sectional descriptive questionnaire survey was undertaken to collect data on CAM attitudes, knowledge and use by physicians. Letters of invitation were sent to 20 institutions, which included: research hospitals, universities and general hospitals, and 11 agreed to participate to the survey. Physicians were invited to complete the questionnaire by the researchers involved in the study (the chief of their department/the chief medical officer). The study was conducted in accordance with the 1964 Helsinki Declaration.

Participants were grouped into four specialty groups: (**G1**) “Oncology/Hematology/Pain management/Radiotherapy/Anaesthesiology” (40.4%); (**G2**) “Internal medicine/Geriatric medicine/Infectious diseases” (25.3%); (**G3**) “Surgical specialties” (15.1%); and (**G4**) Nuclear medicine/No specialty/Other” (19.2%).

### Questionnaire

A 41-item questionnaire was developed by two of the authors (M.B. and A.C.) based on literature data (47) and divided into 3 sections. The first section collected personal and professional data, including participant gender, age, education, medical specialty, years of experience, type of institution and place of work in Italy (North, Center, South and Islands). The second section focused on CAM and asked questions on participants' knowledge of it; their view of its ability to be used with CM; whether they suggest CAM to patients or discuss it with them; whether and how it could be used in their patients, their trust in CM, and their personal use of CAM. The third section asked which CAM interventions were known to the participant; to which patients they would suggest CAM, the role they thought it could have, and which effects they have actually observed. In line with the literature (47), the commonly prescribed medical therapies such as support therapy (e.g., iron, vitamin D, calcium supplements) were not considered as CAM and are not included in the analysis.

### Statistical Analysis

All questionnaires were coded and checked. Missing data and ambiguous responses were excluded from the analysis. Participant information was summarized in descriptive tables. Differences in participant characteristics and knowledge of CAM were analyzed by the chi-square test or Fisher's exact test, as appropriate. The level of significance was set at *p* < 0.05. Analyses were performed with IBM SPSS Statistics 25.0 (49). The variables showing significant differences were entered into a logistic regression model to test the relationships between them (as independent variables) and the four specialty groups, to gain insight into participants' attitudes to CAM. Odds ratios (ORs) and 95% confidence intervals (CIs) were computed to assess participants' attitudes using G1 physicians as the reference category.

## Results

A total number of 438 participants responded, yielding an adjusted response rate of 82% (534 physicians were invited and 96 incomplete questionnaires were excluded). Participants were equally distributed among men and women and their median age was 53 years (range, 30–67). As regards education, 55.7% had a specialization, and only 5% had a Ph.D. degree; the most common specialty areas were G1 (40.4%), G2 (25.3%), G3 (15.1%), and G4 (19.2%); most participants (60.7%) worked in institutions in Southern Italy and were involved in research activity (54%) (Table 2).

**Table 2:** Personal and professional data of participants.

	**No**.	**(%)**
	**438**	**100.0**
**Gender**		
Male	220	50.2
Female	218	49.8
**Age**		
<40 years	189	43.2
40–65 years	239	54.6
65 years	9	2.1
Missing	1	0.2
**Education**		
Medical degree	112	25.6
Medical degree + specialization	224	55.7
Medical degree + specialization + Ph.D.	22	5
Master's degree	12	2.7
Other	48	11
**Specialty group**		
G1	177	40.4
G2	111	25.3
G3	66	15.1
G4	84	19.2
**Years of practice**		
<5 years	118	26.9
5–10 years	70	16.0
> 10 years	209	47.7
Missing	41	9.4
**Institution**		
Research hospital	134	30.6
University	194	44.3
General hospital	110	25.1
**Institution location in Italy**		
North	125	28.5
Center	47	10.7
South and Islands	266	60.7

**G1**: Oncology, Hematology, Pain management, Radiotherapy, Anaesthesiology.

**G2**: Internal medicine, Geriatric medicine, Infectious diseases.

***G3**: Surgical; **G4**: Nuclear medicine, No specialization, Other*.

Slightly more than half (50.9%) knew the meaning of the CAM acronym; most (78.6%) knew about “alternative and complementary medicine,” and most (41.8%) thought that CAM could have a role in CM (Table 3).

**Table 3:** Key questions.

	**No**.	**(%)**
	**438**	**100.0**
**Are you involved in research activity?**		
Yes	240	54.8
No	198	45.2
**Do you know what CAM stands for?**		
Yes	223	50.9
No	215	49.1
**Have you ever heard about alternative and complementary medicine?**		
Yes	342	78.6
No	93	21.4
**Should patients be treated exclusively with CM?**		
Yes	159	36.3
No	194	44.3
I don't know	85	19.4
**Do you suggest CAM to your patients?**		
Yes	241	55
No	197	45
**Do you discuss CAM use with them?**		
Yes	193	44.1
No	212	48.4
I don't know	33	7.5
**Could CAM have a role in CM?**		
Yes	183	41.8
No	100	22.8
I don't know	155	35.4
**Have you seen therapeutic effects of CAM?**		
Yes	203	46.3
No	180	41.1
I don't know	55	12.6
**Yes**		
Psychophysical well-being	95	46.8
Attenuation of treatment side-effects	53	26.1
Improved response rate	11	5.4
Response 1+2	18	8.9
Response 1+3	13	6.4
Response 1+2+3	13	6.4
**Personal use of CAM**		
Yes	112	27.7
No	292	72.3

The statistical comparisons based on specialty group are reported in Table 4. G1 physicians were more likely to work in Northern Italy (34.5%) in a research hospital (42.4%) and were more interested in CAM than the other groups (“Do you know what CAM stands for?” yes, 57.6%; “Have you ever heard about alternative and complementary medicine?” yes, 88%; “Should patients be treated exclusively with CM?” no, 52%). The distribution of physicians involved in research activity and their interest in CAM are reported in Table 5.

**Table 4:** Physicians' characteristics and their CAM knowledge according to their specialty.

**Characteristics**	**Specialty group**						
	**G1 Oncology, hematology, pain management, radiotherapy, anaesthesiology**	**G2 Internal medicine, geriatric medicine, infectious diseases**		**G3 Surgical specialties**		**G4 Nuclear medicine, no specialization, other**	
	**No. (%)**	**No. (%)**	*p*-value 1*	**No. (%)**	*p*-value 2*	**No. (%)**	*p*-value 3*
**Gender**			0.5		0.06		0.2
Female	88 (49.7)	59 (53.2)		24 (36.4)		49 (58.3)	
Male	89 (50.3)	52 (46.8)		42 (63.6)		35 (41.7)	
**Age**			**0.001**		0.6		0.6
<40 years	66 (37.3)	66 (59.5)		29 (43.9)		28 (33.3)	
40–65 years	107 (60.5)	43 (38.7)		35 (53)		55 (65.5)	
65 years	4 (2.3)	2 (1.8)		2 (3)		1 (1.2)	
**Time elapsed since specialization**			**<0.0001**		0.1		0.5
<5 years	39 (23.2)	51 (52.6)		8 (16.7)		20 (23.8)	
5–10 years	36 (21.4)	5 (5.2)		6 (12.5)		23 (27.4)	
≥10 years	93 (55.4)	41 (42.3)		34 (70.8)		41 (48.8)	
**Workplace location**			**<0.0001**		0.8		0.8
Northern Italy	61 (34.5)	17 (15.3)		21 (31.8)		26 (31)	
Central Italy	20 (11.3)	9 (8.1)		7 (10.6)		11 (13.1)	
Southern Italy	96 (54.2)	85 (76.6)		38 (57.6)		47 (56)	
**Institution**			**<0.0001**		**<0.0001**		**0.004**
Research hospital	75 (42.4)	8 (7.2)		15 (22.7)		36 (42.9)	
University	47 (26.6)	72 (64.9)		39 (59.1)		36 (42.9)	
General hospital	55 (31.1)	31 (27.9)		12 (18.2)		12 (14.3)	
**Are you involved in research activity?**			**0.001**		0.9		0.9
Yes	106 (59.9)	44 (39.6)		40 (60.6)		50 (59.5)	
No	71 (40.1)	67 (60.4)		26 (39.4)		34 (40.5)	
**Do you know what CAM stands for?**			**0.01**		0.1		0.3
Yes	102 (57.6)	47 (42.3)		31 (47)		43 (51.2)	
No	75 (42.4)	64 (57.7)		35 (53)		41 (48.8)	
**Have you ever heard about alternative and complementary medicine?**			**<0.0001**		**0.03**		**0.001**
Yes	154 (88)	78 (70.3)		50 (76.9)		60 (71.4)	
No	21 (12)	33 (29.7)		15 (23.1)		24 (28.6)	
**Should patients be treated exclusively with CM?**			0.1		0.06		**0.02**
Yes	58 (32.8)	41 (36.9)		32 (48.5)		28 (33.3)	
No	92 (52)	46 (41.4)		24 (36.4)		32 (38.1)	
I don't know	27 (15.3)	24 (21.6)		10 (15.2)		24 (28.6)	
**Do you suggest CAM to your patients?**			0.2		0.3		0.6
Yes	102 (57.6)	55 (49.5)		33 (50)		51 (60.7)	
No	75 (42.4)	56 (50.5)		33 (50)		33 (39.3)	
**Do you discuss CAM use with your patients?**			0.2		0.1		0.1
Yes	87 (49.2)	51 (45.9)		25 (37.9)		30 (35.7)	
No	74 (41.8)	55 (49.5)		37 (56.1)		46 (54.8)	
I don't know	16 (9)	5 (4.5)		4 (6.1)		8 (9.5)	
**Could CAM have a role in CM?**			**0.005**		0.3		0.09
Yes	86 (48.6)	37 (33.3)		26 (39.4)		34 (40.5)	
No	33 (18.6)	38 (34.2)		18 (27.3)		11 (13.1)	
I don't know	58 (32.8)	36 (32.4)		22 (33.3)		39 (46.4)	
**CAM could play a role as:**			0.9		0.4		0.3
Alternative medicine	11 (6.2)	10 (9)		2 (3)		4 (4.8)	
Complementary therapy	88 (49.7)	55 (49.5)		34 (51.5)		42 (50)	
Integrated medicine	40 (22.6)	26 (23.4)		18 (27.3)		23 (27.4)	
I don't know	38 (21.5)	20 (18)		12 (18.2)		15 (17.9)	
**CAM use by participants**			0.7		0.6		0.7
Yes	43 (25.9)	27 (25.5)		19 (31.7)		23 (31.9)	
No	123 (74.1)	79 (74.5)		41 (68.3)		49 (68.1)	

* *P1; P2; P3: p-values of Pearson's chi-square test comparing G1 physicians with G2 physicians (P1); with G2 physicians (P2); and with G3 physicians (P3)*.

*Bold values indicate statistically significant p-value*.

**Table 5:** CAM knowledge in relation to participants' involvement in research activity.

	**Are you involved in research activity?**		***p*-value**
	**Yes**	**No**	
**Do you know what CAM stands for?**			0.6
Yes	120 (50)	103 (52)	
No	120 (50)	95 (48)	
**Have you ever heard about alternative and complementary medicine?**			**0.01**
Yes	199 (82.9)	143 (73.3)	
No	41 (17.1)	52 (26.7)	
**Are you aware of the difference between complementary and alternative medicine?**			**0.04**
Yes	152 (63.6)	106 (53.8)	
No	87 (36.4)	91 (46.2)	
**Do you suggest CAM to your patients?**			**0.03**
Yes	143 (59.6)	98 (49.5)	
No	97 (40.4)	100 (50.5)	
**Could CAM have a role in CM?**			**0.02**
Yes	109 (45.4)	74 (37.4)	
No	43 (17.9)	57 (28.8)	
I don't know	88 (36.7)	67 (33.8)	
**Do you discuss CAM use with your patients?**			0.1
Yes	108 (45)	85 (42.9)	
No	109 (45.4)	103 (52)	
I don't know	23 (9.6)	10 (5.1)	
**Specialty group**			**0.003**
G1	106 (44.2)	71 (35.9)	
G2	44 (18.3)	67 (33.8)	
G3	40 (16.7)	26 (13.1)	
G4	50 (20.8)	34 (17.2)	

***G1**: Oncology, Hematology, Pain management, Radiotherapy, Anaesthesiology; **G2**: Internal medicine, Geriatric medicine, Infectious diseases; **G3**: Surgical; **G4**: Nuclear medicine, No specialization, Other*.

*Bold values indicate statistically significant p-value*.

CAM knowledge and communication with patients were analyzed by multivariate logistic regression (Table 6). G1 physicians were significantly associated with CAM knowledge (*p* < 0.0001) and with awareness of the difference between complementary and alternative medicine (*p* = 0.01). The lack of an association between G1 physicians and CAM suggestion and prescription to their patients explains their poor propensity for CAM interventions (*p* = 0.4 and 0.09, respectively). About half of participants stated that they do not discuss CAM with their patients.

**Table 6:** Odds ratio (OR) and 95% confidence intervals (CIs) computed to assess the attitudes toward CAM of G1 physicians (specialties: Oncology, Hematology, Pain management, Radiotherapy, Anaesthesiology).

	**G1 vs. G2, G3, G4**	***p*-value**
	**OR**** 95% CI**	
**Do you know what CAM stands for?**		
Yes	1.52 (1.02–2.25)	**0.004**
No	1.00 (Reference category)	
**Have you ever heard about alternative and complementary medicine?**		
Yes	2.64 (1.54–4.52)	**<0.0001**
No	1.00	
**Are you aware of the difference between complementary and alternative medicine?**		
Yes	1.77 (1.18–2.68)	**0.006**
No	1.00	
**Do you suggest CAM to your patients**		
Yes	1.16 (0.79–1.72)	0.4
No	1.00	
**Have you ever prescribed CAM to your patients?**		
Yes	1.47 (0.93–2.32)	0.09
No	1.00	

*Logistic regression model adjusted for age, gender, area of origin and workplace*.

*Bold values indicate statistically significant p-value*.

**Figure 1:**
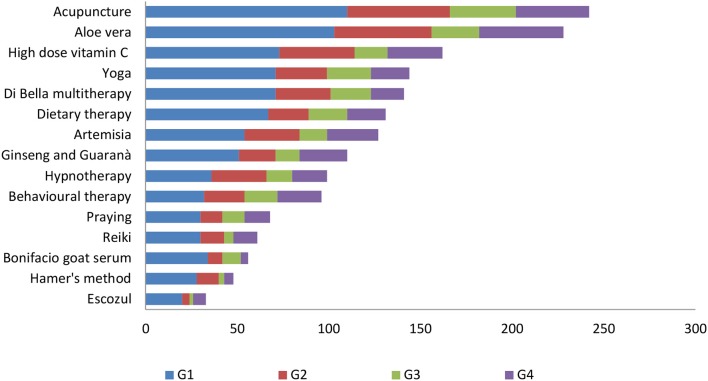
Type of CAM interventions known to participants in relation to specialty group.

The CAM interventions best known to our sample of physicians (Figure 1) were acupuncture (60.7%), *Aloe vera* (57.1%), and high-dose vitamin C (40.6%); the least known were Hamer's method (12%) and *Rophalurus junceus* (poison of the blue scorpion, marketed as “Escozul”) (8.3%). We decided to exclude from this list the medical therapies that are usually prescribed as support therapy (iron, vitamin D, and calcium supplements).

**Figure 2:**
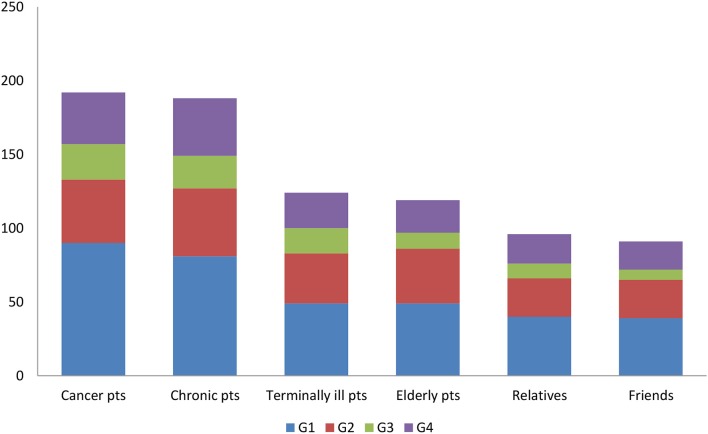
Physicians answer to the question “To whom would you suggest using CAM?”.

The patients to whom participants would recommend CAM therapies (Figure 2) are those with cancer and chronic disease (similar percentages). A significant association was found for none of the specialty groups.

**Figure 3:**
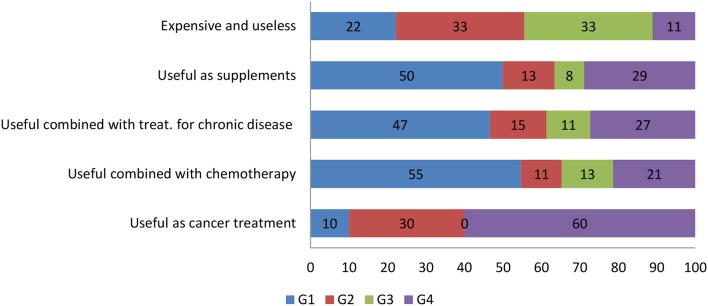
Participants' perception of the value of CAM interventions in relation to their specialty group.

As regards the possible use of CAM (Figure 3), G1 physicians would not recommend their cancer patients to treat their disease with CAM alone (10%) but would recommend it as a support treatment (55%) during AC, whereas G4 physicians would recommend CAM as a ACT (60%). Most (33%) G2 and G3 physicians consider CAM as useless and expensive.

## Discussion

In recent years the interest in CAM has mounted considerably due to media influence and to internet marketing, besides patients' desire to gain greater control on their treatment. The available data suggest that although 29–91% of chronic and cancer patients use CAM remedies together with their treatment, less than half of physicians, and especially of oncologists, discuss them with their patients (4, 5, 7). CAM has largely been ignored by physicians for at least 30 years and has only recently begun to attract the attention of the scientific community and of healthcare institutions.

This study surveyed the CAM knowledge, use, perception, and attitudes of Italian physicians who predominantly treat patients with chronic disease, including cancer. Although 44% of participants believe that patients should be treated exclusively with CM, most (59%) replied that they accept and prescribe CAM interventions. The patients to whom they would prescribe CAM are predominantly those with cancer (76%) or chronic disease (74%) as well as terminally ill (49**%)** and elderly patients (47%). Moreover, 45% (*p* < 0.005) of those surveyed believed that CAM could have a role in CM as a complementary therapy in a context of integrative medicine (IM), as also found by several studies (20, 50–55). Indeed, IM models for cancer patients are applied in hospital setting in several countries (56–59). The analysis of physicians' characteristics highlighted that 40% of our sample are involved in treating cancer patients and work at a university (44%) or a research hospital (31%). Awareness of CAM was acknowledged by 60% of oncologists, by 42% of internal medicine specialists and 45% of “other” specialists; their different knowledge may be due to the widespread use of CAM remedies by cancer patients. This 60% of oncologists constitutes a significant improvement on the 48% described by Crocetti et al. (47) in 1996 and reflects a much greater awareness and knowledge of CAM, a greater attention to the problem and an increased use of CAM in Italy. Participant age (40–65 years) and years of practice (>10 years) were found to be significantly associated with CAM knowledge (respectively, *p* < 0.001 and *p* < 0.002); a similar finding has been reported in a recent national survey of China's oncologists (60). As expected, the physicians with a more limited knowledge of CAM were less likely to discuss it with patients, as also noted by other researchers (61). A recent Norwegian study of cancer patients who use complementary medicine suggests that poor communication experiences with physicians may result in the adoption of CAM interventions, and in some cases in postponement or reduction of the conventional cancer treatment; in contrast, positive communication experiences led to CAM use as a supplement rather than an alternative to CM (62). Effective patient-physician communication may be critical for patient satisfaction and compliance and for favorable outcomes. Patients' negative attitudes toward CM have also been linked to possible adverse reactions to treatment (62). The Norwegian study also reported that patients who had been with the same general practitioner (GP) for more than 2 years were less likely to visit a complementary medicine provider than those with a shorter relationship with their GP (12.5 vs. 15.5%, respectively) (62). Notably, the lack of communication between physicians and providers of complementary interventions is an additional risk for patients who wish to combine what they perceive to be the best of the two worlds.

Interestingly, a study of data from a Dutch health insurance company (63) has found that the patients of GPs who had had CAM training had lower mortality rates and cost less to the healthcare system due to shorter hospital stays and fewer drug prescriptions. In addition, various studies indicate that better educated patients with higher than average incomes are more likely to choose CAM and are frequently supported in this choice by their GPs (7, 64–66). Informing physicians about the high prevalence of CAM use and the commonly used CAM interventions has the potential to advance communication with patients. Our survey found that half of physicians discuss the role of CAM with their patients: these physicians are those who are involved in research work, have more than 10 years of specialization and belong to G1 group.

The need for improving physicians' CAM knowledge and communication with patients has also been highlighted in recent studies by the Working Group Prevention and Integrative Oncology of the German Cancer Society (54), the German society for Palliative Medicine (67), other German institutions (68, 69) and the national survey of China's oncologists (60). The German studies also indicate that some CAM practices (psycho-oncology, sport, micronutrient supplements) are more popular in Germany than in Italy.

Negative experiences related to physician-patient interactions and CM outcomes can encourage cancer patients to use CAM and to refuse or postpone CM (70).

In our survey, the physicians working at a university and/or a research hospital knew CAM significantly better than those who worked at a general hospital (*p* < 0.0001), and those who were also involved in research work knew CAM better than those who did no research (*p* < 0.003). Similar results are reported in the national survey of Chinese oncologists: those working in metropolitan areas and academic hospitals have a greater knowledge of and a more favorable attitude toward CAM (60). Interestingly, in our survey 60% of the physicians involved in research would suggest CAM to patients, and 45% of them discuss it with them; surprisingly, this is also the proportion of physicians who do no research (*p* < 0.1). Our survey demonstrated that the lack of communication about CAM between physicians and patients is not necessarily related to physicians's knowledge of CAM. The CAM interventions best known to participants were acupuncture (60.7%), *Aloe vera* (57.1%), high-dosage vitamin C (40.6%), and yoga (36.1%), whereas the least known was Escozul (8.3%). Surgeons were the physicians with the most limited CAM knowledge. All physicians stated they would prescribe CAM chiefly to patients with cancer and/or chronic disease; 33% of internal medicine physicians feel that CAM is useless and expensive, 50% of oncologists think that CAM remedies could be used as supplements, and 47% of them consider CAM useful as support in chronic treatments. Notably, most (55%) G1 physicians view CAM as a support treatment during ACT and only 10% believe that it can be used as an ACT. Interestingly, 30 and 60% of G2 and G4 physicians, respectively, believe that CAM can have a role as an ACT. Such widely different views could be related to lack of CAM training in the medical degree course. Similar to our oncologists, the national survey found that China's oncologists accept CAM (44.9% of participants) to manage the most common symptoms related to cancer treatment such as lack of appetite, fatigue and sleep disorder (60), i.e., as support treatment. Moreover, 22% of G1 and 33% of G2 and G3 physicians consider CAM expensive and useless. To improve CAM knowledge, most U.S. medical schools (64%) are offering alternative medicine courses (69, 71). Moreover, a recent study has reported that 95% of students in an Arabic medical school were satisfied with a course on integrative and prophetic medicine (72). These data indicate an increasing need for greater insight into CAM interventions, mostly for use with CM.

The two chief limitations of the study are the size of the sample and the fact that an interest in CAM may have enhanced respondents' willingness to participate. However, this the first survey involving a large number of physicians of several specialties, all of whom are involved in treating patients with chronic conditions, including cancer. Moreover, analysis of their responses, to highlight different approaches to CAM, enabled extensive dissection of the data, since participants were grouped into specialty groups as well as by their involvement in research work and the type and geographical site of their institution.

In conclusion, our survey provides up to date information about physician's knowledge of CAM and their attitudes to it. The CAM awareness of Italian physicians has considerably improved since the late 1990's, when a similar questionnaire was distributed, and their attitudes have changed accordingly. Although it is difficult to assess their CAM knowledge, attitudes and practice patterns and their true prevalence, we believe that this survey provides new and topical information. Since in Italy the question is increasingly being discussed by the medical and the lay community alike, this study provides a long overdue update on a highly topical issue.

## Perspectives

The lack of CAM knowledge by physicians and their limited communication with patients have negative consequences on and implications for clinical management and outcomes. Notably, it has been demonstrated that the use of CAM instead of CM was associated with worse five-year survival in cancer patients (73). The use of CAM by cancer patients is therefore an outstanding issue that warrants greater attention by the scientific community and physicians. Critically, its unguided use by patients with chronic disease and/or cancer has important implications for healthcare services and care providers as well as for the patients themselves. Assessing the soundness of CAM information sources and improving communication with physicians on this topic is crucial to enhance or preserve patient health and to strengthen the therapeutic relationship and patient compliance. We believe that physicians should expand their knowledge of CAM interventions, beneficial effects and potential interactions and toxicity. Indeed, an earlier pilot study (74) has identified 47 different potential interactions among 136 herb-drug combinations whereas a more recent investigation has found that 37.2% of patients were at risk of interaction between CM and CAM interventions (75). This risk can be reduced by improving physician-patient communication, as shown by several studies (50–55, 76, 77), as well as by the adoption of an integrative medicine model. It would be useful to run clinical trials on some interventions, like mushrooms, mistletoe, ozone, and high-dose vitamin C, for which there is some scientific evidence (78–87). It is essential to find an evidence base for CAM therapies using suitable, sensitive approaches. Discussion of CAM interventions and guidance on potential benefits and toxicities is a task that physicians should urgently undertake. Extensive research is required to assess actual CAM use and dosage in patients receiving different treatments and to work toward achieving an integrated model of healthcare provision, which should also inform EU legislation.

## Data Availability Statement

The datasets generated for this study are available on request to the corresponding author.

## Author Contributions

MB, RT, RD, ACr, and GF conceived the study. MB, LR, RT, PT, RD, GP, GNu, ACr, and GF developed the study design. MB, PG, MC, ACr, and GF oversaw the study. MB, PG, MC, RD, ACu, and GF drafted the manuscript. MB, PG, and ACr analyzed the data. All authors have read and approved the final manuscript.

## Conflict of Interest

The authors declare that the research was conducted in the absence of any commercial or financial relationships that could be construed as a potential conflict of interest.
